# The Relationship Between Problematic TikTok Use and Depression in University Students: The Mediating Role of Insomnia

**DOI:** 10.3390/jcm14134652

**Published:** 2025-07-01

**Authors:** Aleksandra M. Rogowska, Olga Lechowicz

**Affiliations:** Institute of Psychology, Faculty of Social Sciences, University of Opole, Staszic Square 1, 45-052 Opole, Poland

**Keywords:** depression symptoms, medical and healthcare faculties, gender differences, insomnia, problematic TikTok use, university students

## Abstract

**Background/Objectives**: University students are particularly susceptible to mental health issues, exhibiting a higher prevalence of insomnia and depressive symptoms compared to the general population. These mental problems adversely affect their academic performance and overall well-being. Understanding this issue is essential for developing effective prevention and intervention strategies for the academic community. The present study investigates the complex role of problematic TikTok use and insomnia in relation to depression among university students. **Methods**: An online cross-sectional survey was conducted between November 2024 and January 2025, involving a sample of 173 university students in Poland. The participants had a mean age of 23 years (*M* = 23.09, *SD* = 3.92), with 73.4% being women, and 49% were enrolled in medical and healthcare faculties. The questionnaire comprised a demographic survey, the Patient Health Questionnaire (PHQ-9) for assessing symptoms of depression, the Athens Insomnia Scale (AIS-8) for measuring symptoms of insomnia, and the modified Bergen Facebook Addiction Scale (BFAS) for evaluating problematic TikTok use (PTTU). The hypotheses were verified using Student’s *t*-test, Pearson’s correlation, and general linear model (GLM) mediation analysis. **Results**: The study found no significant gender (women vs. men) and faculty (medical vs. non-medical) differences in the level of symptoms of depression, insomnia, or problematic TikTok use. Insomnia, depression, and PTTU were positively inter-related. Insomnia completely mediates the relationship between PTTU and depression in university students. However, these results must be treated with caution due to the uneven gender distribution and cross-sectional nature of these studies, which limits causal relationships. **Conclusions**: Excessive engagement with TikTok has been associated with an increase in insomnia symptoms, which subsequently exacerbates depression symptoms among university students. Prevention and intervention strategies should prioritize reducing TikTok usage while simultaneously enhancing sleep hygiene and mental health within the academic population, irrespective of gender and university faculty affiliation.

## 1. Introduction

### 1.1. Insomnia Among University Students

Insomnia is a common sleep problem characterized by difficulties with sleep initiation, maintenance, early awakening, and nonrestorative sleep, often leading to significant daytime problems. Core symptoms of insomnia can be divided into two dimensions: (1) nighttime symptoms: difficulty falling asleep (sleep onset insomnia), trouble staying asleep (sleep maintenance insomnia); (2) early morning awakening, and nonrestorative sleep (waking up feeling unrefreshed) [1,2]; and daytime impairments: fatigue, reduced attention, impaired cognitive functioning, irritability, anxiety, low mood, and diminished quality of life during the day [1,3]. Insomnia disorder is diagnosed primarily through self-report, focusing on both nighttime and daytime symptoms [1,4]. Insomnia symptoms can occur alone or alongside other medical or psychiatric conditions, and they are associated with a range of adverse health outcomes.

Insomnia symptoms are a significant concern among university and college students, with research showing high prevalence rates and essential links to mental health, academic performance, and lifestyle factors [5,6,7,8]. Understanding the patterns and predictors of insomnia in this population is crucial for effective prevention and intervention. A systematic review highlighted that the rate of insomnia among university students is significantly higher compared to the general population, with figures at 18.5% and 7.4%, respectively [9]. More recent studies showed that the prevalence rates of insomnia symptoms among university students range from about 16% to nearly 40% [10,11,12,13,14,15]. These rates have increased during the COVID-19 pandemic. For example, over half of Polish university students reported insomnia symptoms during the pandemic [16,17]. In addition, medical students may be even more vulnerable to sleep disturbances compared to other student groups [12,18]. All of the findings mentioned above indicate that greater attention should be directed towards addressing insomnia in university students, especially medical and healthcare students.

Insomnia symptoms not only affect well-being but also academic performance, highlighting the need for targeted prevention and support strategies in this population. University students who have chronic insomnia often experience lower academic achievement and engage in unhealthy behaviors [11]. Also, fatigue, restlessness, and irritability are common symptoms that bridge insomnia, depression, and anxiety among students [19]. Insomnia symptoms are significantly related to mental disorders, such as depression and anxiety, in university and college students [11,12,13,15,20,21,22,23].

### 1.2. Depression in University Students

Depression is a mental health disorder marked by persistent feelings of sadness, emptiness, or irritability, along with a range of physical and cognitive symptoms that significantly impact daily functioning. The presentation of depression can vary widely between individuals, and symptoms must be present nearly every day for at least two weeks to meet diagnostic criteria. Core symptoms of depression are experienced in each of four domains of psychological functioning: (1) emotional (i.e., persistent sadness, emptiness, hopelessness, irritability, self-hatred, loneliness, and pessimism); (2) cognitive (e.g., difficulty concentrating, indecisiveness, negative self-image, self-blame, and thoughts of worthlessness or guilt); (3) physical (i.e., changes in appetite, sleep disturbances including insomnia or hypersomnia, fatigue, low energy, headaches, unexplained aches and pains, and digestive problems); and (4) behavioral (e.g., anhedonia, which refers to a loss of interest or pleasure in activities, withdrawal from social interactions, and changes in activity levels either agitation or slowed movements) [24,25,26]. Depression is highly heterogeneous, with at least 52 different symptoms identified across common depression scales [26]. Many symptoms appear on only one scale, and there is a low overlap between different measurement tools. In young people, self-hatred, loneliness, sadness, and pessimism are especially central, with strong links between sadness and crying, anhedonia and school dislike, and sleep disturbance and fatigue [24]. Even after treatment, many patients experience lingering symptoms such as fatigue, anxiety, sexual dysfunction, and sleep disturbances, which can affect long-term outcomes [27].

Depression is a significant mental health concern among university students worldwide, with medical and healthcare students often experiencing even higher rates due to academic and emotional pressures. The prevalence of depression among university students varies widely, with estimates ranging from 10% to 39.5% across different countries and studies [28,29,30,31,32]. A comparison of mental health between three UE countries during the COVID-19 pandemic showed that 43% of Polish and German university students suffer depression symptoms, while among Slovenian students, these rates were significantly lower, indicating 25% [33]. A substantial proportion of students experience moderate to severe depression, with some studies reporting up to 15.6% suffering from severe or extremely severe symptoms [31,34]. Subgroup analyses indicate that medical students have a higher prevalence of depression compared to students in other fields [35]. Among Polish medical students [36], 51.61% reported experiencing symptoms of major depression, with 26.00% exhibiting moderate symptoms, 15.05% moderately severe symptoms, and 10.56% severe symptoms. Additionally, 30.21% of the participants reported mild depression symptoms [36]. Depression often co-occurs with anxiety and stress, further impacting students’ well-being and academic performance [31,33,37,38,39,40]. Among the various factors contributing to the decline in insomnia and depression, the excessive use of social media emerges as a significant attribute of the modern lifestyle.

### 1.3. The Role of Social Media Use in Increasing Symptoms of Insomnia and Depression

Problematic use of social media is increasingly recognized as a behavioral problem characterized by excessive, compulsive use of social networking platforms, leading to negative impacts on daily life, relationships, and well-being [41,42]. Research highlights a complex interplay of psychological, social, and technological factors driving this addiction, especially among adolescents and young adults [42,43,44,45]. Individuals experiencing problematic social media use may find it challenging to control the amount of time spent on social media platforms, leading to neglect of other vital aspects of their lives [46]. Key symptoms and characteristics include the following: (1) excessive use (i.e., persistent, uncontrollable urge to check or use social media, often resulting in excessive screen time and neglect of real-life responsibilities); (2) compulsive behavior (i.e., repeated, habitual checking of platforms, even when it interferes with work, study, or social interactions); (3) mood modification (i.e., use of social media to escape negative emotions, relieve stress, or alter mood, sometimes leading to a cycle of dependence); and (4) withdrawal and tolerance symptoms (feelings of irritability, anxiety, or discomfort when unable to access social media and needing to spend increasing amounts of time online to achieve the same satisfaction) [47,48,49,50,51]. Social media addiction can lead to reduced productivity, unhealthy social relationships, lower life satisfaction, and potential mental health issues [52,53,54]. TikTok is a social media platform that focuses on the creation, sharing, and exploration of short videos. It is recognized for its easy-to-use interface, a wide range of content, and popular trends, and it is owned by ByteDance, a technology company based in China. TikTok use is widespread among adolescents and young adults, such as university students, reflecting broader trends in social media engagement [55]. The platform allows users to express themselves, showcase talents, and participate in trends, which fosters creativity and engagement [56,57,58]. Students report positive attitudes toward using TikTok for academic purposes, finding it innovative and engaging [59,60]. Despite positive aspects, TikTok use can also trigger feelings of insecurity, social anxiety, and self-comparison [42,44,45]. Factors such as perfectionism and exposure to idealized content may negatively affect students’ mental well-being, highlighting the need for strategies to manage these effects [61]. Recent systematic research has demonstrated an association between TikTok usage and increased isolation and hopelessness, which, in turn, exacerbate symptoms of anxiety and depression, with several factors contributing to problematic TikTok use, including maladaptive behavioral patterns, some demographic factors (i.e., young age, female sex, and low socioeconomic backgrounds), and certain personality traits (such as high procrastination and neuroticism along with low self-control and tolerance of distress) [62,63]. Excessive TikTok use can lead to reduced sleep time at night and increased sleepiness at work/during study [64]. Furthermore, TikTok has been identified as a source of mental health information but also misinformation, which may increase the risk of mental disorders [62].

Studies indicate that excessive use of the Internet and smartphones, particularly for social media, significantly impacts daily life by heightening stress, depression, and insomnia among young adults, including university students [5,44,45,61,65,66,67,68,69,70,71,72,73,74]. In particular, university students who use their phones for more than two hours daily have a significantly higher risk of both insomnia and depression compared to those with less usage [67]. However, the role of TikTok in mental health outcomes should be better explained, as this application is relatively new. In particular, there is a lack of research on the university student population that would include measuring several dimensions of mental health and examining TikTok. The current study focuses on the mental health challenges faced by university students, particularly in relation to problematic TikTok usage, insomnia, and depression. The prevalence of these issues has been exacerbated by the COVID-19 pandemic, which significantly disrupted students’ daily routines and social interactions. As a result, many students have turned to social media platforms like TikTok for entertainment and connection, potentially leading to excessive use and negative mental health outcomes. This research aims to investigate the relationships between problematic TikTok usage, insomnia, and depression among university students, with the goal of identifying potential interventions and support strategies.

### 1.4. The Current Study

University students are at the highest risk of mental health disturbances compared to the general population [7]. The transition to university life presents significant challenges for students. Factors like heightened stress, academic pressures, and social adjustments can contribute to these mental health challenges [5,6,8,21,23,75]. University students encounter various stressors, including an increased academic workload, heightened responsibilities and independence, and the necessity of cohabitating with fellow students. These factors can contribute to a heightened risk of experiencing irregular sleep patterns and poor sleep quality, which may exacerbate symptoms of anxiety and depression [7,8,76]. The excessive use of social media platforms, such as TikTok, constitutes an additional risk factor for the deterioration of well-being among university students. The current research endeavors to clarify the impact of TikTok engagement and insomnia on depressive symptoms in university students.

This research seeks to explore the intricate relationship between excessive TikTok usage, sleep disturbances, and symptoms of depression in university students. Excessive or maladaptive engagement in the TikTok platform may be associated with increased difficulty falling asleep or maintaining sleep quality, as well as increased experiences of depressive symptoms. This proposed relationship suggests that students who exhibit problematic TikTok use patterns may be more susceptible to both sleep and mood disorders, potentially affecting their overall well-being and academic performance.

**Figure 1:**
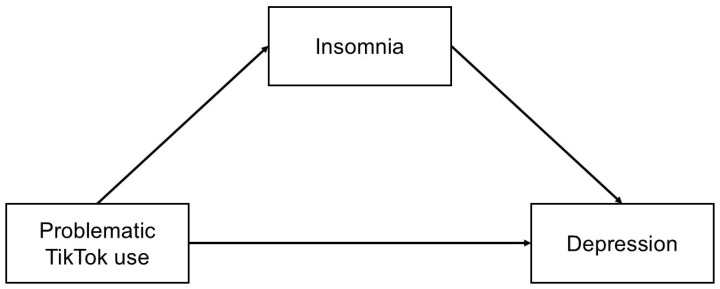
Hypothetical mediation model to explain the indirect effect of problematic TikTok use on depression via insomnia symptoms.

The study aims to elucidate potential mechanisms through which problematic TikTok use may affect depressive symptoms. By examining the mediating role of insomnia, the study aims to discover whether sleep disorders act as a key mediating factor in the association between TikTok use and depressive symptoms. This approach acknowledges the multifaceted nature of mental health and sleep problems, recognizing that the impact of social media use on mental well-being may involve both direct and indirect pathways. Understanding these relationships may provide valuable information for developing targeted interventions and strategies to promote healthier social media habits and improve sleep quality among college students. Li et al. [77] found that insomnia partially mediates the relationship between problematic Internet use and depression among secondary school students. However, this multifaced association has not yet been explored among university students, especially those at higher risk of mental health problems such as women and students of medical and healthcare faculties. Previous research has indicated that university students who engage in excessive smartphone use, particularly on social media platforms, are significantly more likely to experience insomnia and depression [67,68,69,70,71]. However, little is known about TikTok use among medical and healthcare students and its impact on mental health outcomes. In this research, we will examine, for the first time, how insomnia mediates the link between problematic TikTok usage and depression in university students. Based on the previous literature, we hypothesize that women and students representing medical and healthcare faculties demonstrate higher levels of problematic TikTok use, insomnia, and depression than men and students of other faculties (Hypothesis 1 [H1]). We assume that problematic TikTok use correlates positively with insomnia and depression symptoms and that high insomnia levels are related to high depression symptoms in university students (Hypothesis 2 [H2]). Finally, we hypothesize that problematic TikTok use contributes to depression symptoms directly, as well as indirectly via insomnia symptoms (Hypothesis 3 [H3]). We assume that insomnia plays a crucial mediating role as one pathway through which TikTok overuse leads to increased depressive symptoms (Figure 1).

This study also conducts a sensitivity analysis to examine the differences in insomnia and depression among university students who exhibit problematic TikTok use compared to their peers without symptoms of social media addiction. We hypothesize that students who excessively use TikTok will demonstrate higher levels of insomnia and depression symptoms than those who do not engage in problematic TikTok use (Hypothesis 4 [H4]). This investigation aimed to confirm the previously stated hypothesis (H2) about potential correlations between excessive TikTok usage and mental health outcomes. By comparing these two groups, research can gain deeper insights into the potential impact of problematic social media engagement on sleep patterns and mood disorders among young adults in academic settings.

## 2. Materials and Methods

### 2.1. Study Design and Procedure

The cross-sectional online survey was conducted via Google Forms from 18 November 2024 to 26 January 2025. Participants were recruited through private groups on social media platforms, including Facebook and Instagram. The inclusion criteria required the participants to be at least 18 years old and to be university students. The University Committee for Research Ethics at the University of Opole approved the research protocol (Decision No. 53/2024, 12 December 2024). The study was undertaken as part of a master’s thesis by one of the authors (OL). The application to the Ethics Committee at the University of Opole was submitted three months before the study started. To avoid delaying the completion of the master’s degree at the Institute of Psychology, the authors proceeded with the study. Therefore, the decision was rendered only during the data collection phase. However, it is important to note that the authors prior ensured that all ethical guidelines were adhered to during data collection, in accordance with the Declaration of Helsinki and the research standards recommended by the American Psychological Association (APA). Participation in the study was voluntary and anonymous. Informed consent was required before taking part in the study. Responses to all questions were mandatory; therefore, there were no missing data in this study.

An a priori power analysis was conducted using G*Power version 3.1.9.7 [78] for sample size estimation. Considering the medium effect size (Cohen’s *d* = 0.50), a significance criterion of α = 0.05, and power = 0.80, the minimum sample size needed with this effect size is *N* = 102 for the independent samples Student’s *t*-test. For correlation analysis, *N* = 67 is required, while for linear regression analysis with two predictors, *N* = 67 is expected. Initially, 175 individuals responded to the invitation; however, 2 declined to participate in the survey study. Consequently, the final sample size of *N* = 173 is sufficient to test the study hypothesis.

### 2.2. Measures

#### 2.2.1. Insomnia

The Athens Insomnia Scale (AIS-8) is an 8-item tool to measure insomnia symptoms based on the International Classification of Diseases (ICD-10) criteria [79,80]. Each item is rated by the respondent on a scale of 0–3 points, where 0 means the absence of a given symptom, and 3 denotes its significant severity. The total score on the scale is between 0–24 points. A score of 6 or above is typically seen as a sign of insomnia. The reliability was appropriate in the Polish version of the AIS-8 (Cronbach’s α = 0.90) [81], as well as in the present study (Cronbach’s α = 0.86).

#### 2.2.2. Depression

The nine-item Patient Health Questionnaire (PHQ-9) is a screening tool comprising particular symptoms of depression [82]. Respondents are asked to indicate the frequency with which they have experienced each symptom over the past two weeks, using a 4-point scale ranging from 0 (not at all) to 3 (nearly every day) points. The total score ranges from 0 to 27, with higher scores indicating greater severity of depressive symptoms. A score of 10 serves as the threshold for moderate-to-severe depression symptoms. The Cronbach’s alpha for the Polish version is α = 0.88 [83], while in the present study, it is α = 0.83.

#### 2.2.3. Problematic TikTok Use

Problematic TikTok use (PTTU) was assessed using the modified Bergen Facebook Addiction Scale (BFAS) [50]. The BFAS was initially created to assess problematic Facebook usage, but it was soon adapted for various social media platforms, including Instagram, Twitter, and TikTok [84,85]. The BFAS comprises six items, each delineating a fundamental aspect of behavioral addiction: salience, mood modification, tolerance, withdrawal, conflict, and relapse. In the context of this study, it was imperative to substitute the term “Facebook” with “TikTok” in the questionnaire items. Participants evaluate the frequency of the specified behavior on a five-point Likert scale, ranging from 1 (very rarely) to 5 (very often). The total score ranges from 6 to 30, with higher scores indicating an increased risk of social media addiction. A cut-off score of 18 is used to denote problematic use of social media [50]. Reliability in the Polish version of the PTTU was Cronbach’s α = 0.86 [55], and in the present study, α = 0.89.

#### 2.2.4. Demographics

The demographic survey included age (number of years), gender (woman, man, or other), relationship status (single or in a relationship), place of residence (village, town, or city), living arrangement (I am renting a room/apartment where I study, I live in a flat/house in the city where I study, I live in a dormitory, I live in a town other than the city where I study, and I commute), working status (I work full time/over 20 h a week, I work part time or casually (additional work, on weekends, less than 20 h per week, I do not work, but I support myself with a scholarship (academic scholarship, sports scholarship, etc., I do not work and support myself in other ways, e.g., savings and support from loved ones)), economic status (I am satisfied with my economic status or I am not satisfied with my economic status), type of study (full time or part time), year of the study (ranging from 1 to 6), and faculty of study (medical or healthcare field: medical studies, pharmacy, midwifery, nursing, cosmetology, and medical rescue; non-medical field: pedagogy, computer science, English language studies, journalism, physics, etc.).

### 2.3. Participants’ Characteristic

The study comprised 173 participants, specifically Polish university students, categorized into medical (*n* = 84) and non-medical (*n* = 89) fields. Among the participants, 127 were female (73.4%), 45 were male (26%), and 1 individual opted not to disclose their gender (0.06%). The age of the participants ranged from 18 to 50 years, with a mean age of 23 (*M* = 23.09, *SD* = 3.92). A majority, 79.2%, were full-time students (*n* = 137), while 20.8% were part-time students (*n* = 36). Regarding academic standing, 32.4% were in their fifth year (*n* = 56), 24.3% in their second year (*n* = 42), 16.2% in their fourth year (*n* = 28), 15% in their third year (*n* = 26), 9.2% in their first year (*n* = 16), and 2.9% in their sixth year (*n* = 5). In terms of relationship status, 61.3% reported being in a relationship (*n* = 106), while 38.7% identified as single (*n* = 67). More than half of the respondents (50.9%) originated from cities (*n* = 88), 29.5% from towns (*n* = 51), and 19.7% resided in villages (*n* = 34). The majority of participants rented accommodation in the city where they studied (*n* = 80), 30.1% commuted (*n* = 52), 17.3% lived in a house or owned an apartment in the city of study (*n* = 30), and 6.4% resided in dormitories (*n* = 11). Regarding employment, 33.5% did not work and relied on savings or familial support (*n* = 58), 32.9% engaged in casual work (*n* = 57), 25.4% were employed full time (*n* = 44), and 8.1% depended on scholarships (*n* = 14). A significant proportion of respondents (68.2%) expressed satisfaction with their economic status (*n* = 118).

### 2.4. Statistical Analysis

A preliminary descriptive analysis was performed to examine parametric properties of depression, insomnia, and PTTU in the university student sample. Since the sample size was quite large (*N* = 173) and skewness and kurtosis ranged between −1 and +1, the parametric tests were implemented for the next steps, as the data distribution was not significantly different from normal distribution. To examine how depression, insomnia, and PTTU differ across genders and faculty samples, the independent samples Student’s *t*-test was performed. In addition, Student’s t-test was used to examine differences in insomnia and depression symptoms between students who used TikTok excessively and non-problematically. Pearson’s correlation was conducted to examine associations between depression, insomnia, and PTTU in the university student sample. Finally, the general linear model (GLM) mediation analysis investigates how PTTU influences depression through insomnia as the mediator. The module estimates simple, multiple, and conditional mediation models with maximum likelihood regression. The confidence intervals (CI) for estimates are computed using the bias-corrected bootstrap method with 1000 sample replications. All statistical analyses were performed using JAMOVI ver. 2.6.24 for Windows.

## 3. Results

### 3.1. Intergroup Differences in Depression, Insomnia, and Problematic TikTok Use Among University Students

Among the university student cohort, 48% (*n* = 83) met the criteria for depressive symptoms, as indicated by a PHQ-9 score equal to or greater than 10. Additionally, 65% (*n* = 114) exhibited symptoms of insomnia, with an AIS score equaling or exceeding 6, and 22% (*n* = 39) demonstrated problematic TikTok use, as evidenced by a PTTU score equal to or greater than 18. The independent samples Student’s *t*-test was performed to examine gender and faculty differences among participants. The study found no significant differences in the levels of symptoms of depression, insomnia, or problematic TikTok use, neither by gender (Table 1) nor by faculty (Table 2).

As a sensitivity analysis, differences between university students using TikTok non-problematically (*n* = 134) and problematically (*n* = 39) were examined using the independent samples Student’s *t*-test (Table 3). University students who use TikTok problematically exhibit significantly higher symptoms of insomnia and depression than their peers who use TikTok moderately, with a medium effect size.

### 3.2. Association Between Depression, Insomnia, and Problematic TikTok Use

**Figure 2:**
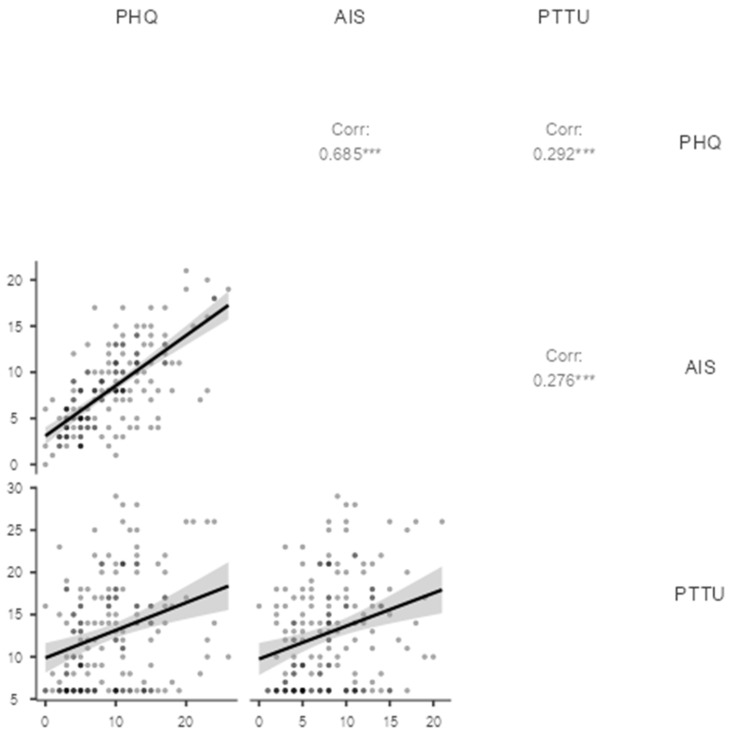
Pearson’s correlation between depression symptoms (PHQ), insomnia (AIS), and problematic TikTok use (PTTU). *N* = 173. *** *p* < 0.001.

Pearson’s correlation analysis showed that depression symptoms are related positively to insomnia symptoms (*r* = 0.69, *p* < 0.001) and problematic TikTok (*r* = 0.29, *p* < 0.001) use among students (Figure 2). In addition, a positive correlation was found between insomnia symptoms and problematic TikTok use (*r* = 0.28, *p* < 0.001).

**Figure 3:**
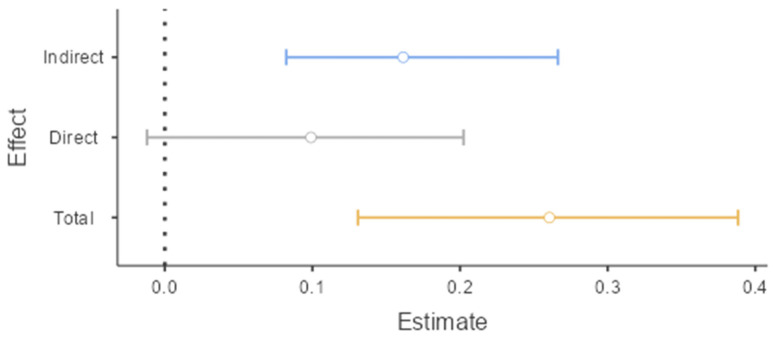
Estimate plot for the mediating effect of insomnia on the relationships between problematic TikTok use and depression (*N* = 173).

The mediation role of insomnia on the relationships between problematic TikTok use and depression was examined using GLM mediation analysis (Table 4). The total effect of PTTU on depression was significant (β = 0.29, *p* < 0.001). When insomnia was included in the mediation model, PTTU was a significant predictor of insomnia (β = 0.28, *p* < 0.001), and insomnia was a significant predictor of depression (β = 0.66, *p* < 0.001). However, the association between PTTU and depression was no longer significant (direct effect), indicating that insomnia completely mediates the relationships between PTTU and depression among university students. The indirect effect of PTTU on depression via insomnia symptoms was significant (β = 0.18, *p* < 0.001). Figure 3 demonstrates bootstrap confidence intervals for estimates of indirect, direct, and total mediation effects. Overall, the mediation model explained 48% of depression variability, with *R*^2^ = 0.48, *F*(2, 170) = 78.84, and *p* < 0.001.

## 4. Discussion

### 4.1. Prevalence of Insomnia, Depression, and Problematic TikTok Use

The study explored, for the first time, the complex relationships between insomnia, problematic TikTok use, and symptoms of depression in university students. Gender and faculty (medical and healthcare faculties) were controlled in the study. The research indicated that a high rate of university students suffering from depression (48%) and insomnia (65%) symptoms. Problematic TikTok use was found in 22% of the sample, confirming previously found evidence. For example, the potential prevalence rate of Internet addiction was 16.8% among medical university students from Lebanon [86].

The prevalence of mental problems in the present study is similar to those previously referred to. For example, 54.1% of Polish university students had insomnia, as indicated by the AIS scores, and 26.1% displayed sleepiness during the day [17]. Similarly, over half of the sample of Polish university students had some form of sleep disturbance during the COVID-19 pandemic, with moderate-to-severe insomnia symptoms noted in 21.6% [16]. However, these rates varied between 16% and 40% across various studies depending on faculty and measurement methods [9,10,11,12,13,14,15]. Among Polish medical students, insomnia was reported by 36.8% of respondents [18]. In one large sample, 16.4% [10] had clinical-range insomnia symptoms, while another study found 39.7% of students reported insomnia symptoms [11]. The inconsistency between particular studies may be determined by various methods of diagnosis (various self-report questionnaires or clinical interviews), cut-off points, various criteria of insomnia, and intercultural differences. Therefore, more research is needed to examine the inconsistency between particular studies regarding the prevalence of insomnia among university students.

Studies have identified several key factors contributing to the increase in insomnia symptoms among students, including poor sleep hygiene, elevated screen time (particularly on weekends and for activities such as video gaming or productivity), and the use of mobile devices before bedtime [10,11,13]. Among demographics and psychological factors, being female and of older age [13], as well as having a high level of perfectionism, low self-esteem, and an external locus of control, are linked to greater insomnia symptoms [20]. Also, evening chronotype, psychological distress, and stimulant or sleep medication use were found as predictors of insomnia, particularly in male students [14].

Also, depression is highly prevalent among university students. Previous research indicated that depression symptoms are present in 10% to 43% of university students in various countries [28,29,30,31,32,33,38]. The present results are even higher but similar to studies performed during the COVID-19 pandemic among young Polish and German adults [33]. Academic stress, personal and social factors, and demographic characteristics all contribute to this elevated risk. Addressing these factors through targeted mental health support and campus interventions is essential for improving student well-being. Research indicates that increased depressive symptoms are linked to such risk factors as high academic demands, perceived adverse academic environments, and semester timing (with peaks in stress and depression around exam periods) [34,87]. Also, a high sense of loneliness, low physical activity, regular binge drinking, and less body appreciation are associated with higher depression rates [31,37]. In contrast, students with positive views of their university environment and supportive living arrangements report lower depression levels [34].

### 4.2. Gender and Faculty Differences in Insomnia, Depression, and Problematic TikTok Use

In contrast to our hypothesis (H1), no gender and faculty differences were found in the levels of depression, insomnia, and PTTU among university students. However, it is important to note that gender was not evenly distributed, and women significantly predominated in this group of students. Therefore, these results should be treated with caution. Previous research showed that medical students demonstrated higher rates of depression and sleep disorder symptoms than their counterparts in other faculties [12,18,88]. The present study seems to be in line with our previous research, which showed no gender differences in PTTU and depression symptoms among emerging and young adults [55]. However, other studies consistently demonstrated that female university students have higher levels of social media addiction, insomnia, and depression symptoms than their male counterparts [17,38,86,88]. For example, Babicki et al. [17] showed that female university students more often suffered from sleep disorders than males. Younes et al. [86] found gender differences in problematic use of the Internet, with males exhibiting a higher prevalence than females (23.6% vs. 13.9%, respectively). Regarding gender as a risk factor, some studies find higher depression rates among male students, while others report no significant gender difference [29,33,37,38,39,88,89].

It is important to note that several other factors may also contribute to mental health outcomes in the population of university students. Single students tend to have higher depression rates than those in romantic relationships [29,33,38]. Parental education and financial struggles are also significant correlates [39,40]. Among Polish medical students, a statistically significant association was identified between major depression and several factors: frequent feelings of loneliness, lack of regular physical activity, insufficient sleep, infrequent participation in social gatherings, difficulties in maintaining stable body weight, alcohol use for stress or negative emotion relief, and non-religious affiliation [36]. It is impossible to control all of the potential risk factors, but future studies should take into account more variables to explain the results of the present study.

### 4.3. Associations Between Insomnia, Depression, and Problematic TikTok Use

Consistent with our hypothesis (H2) and previous studies [67,68,69,70,71], we found positive correlations between all variables: PTTU, insomnia, and depression. The exploration of differences in insomnia and depression among university students with problematic TikTok use versus those without social media addiction symptoms serves as an additional analysis. The hypothesis (H4) that students overusing TikTok simultaneously exhibit higher levels of insomnia and depression symptoms compared to their counterparts who use the platform non-problematically was supported in the study. This comparison revealed important patterns in sleep disturbances and depressive tendencies associated with excessive TikTok use. Such findings could contribute to a broader understanding of the relationship between social media habits and mental well-being in university populations, potentially informing interventions and support strategies for students struggling with problematic social media use.

Previous research has demonstrated that university students exhibiting elevated symptoms of Internet addiction also experience increased symptoms of insomnia and depression [86]. Much evidence showed that insomnia and depressive symptoms are closely linked, with several psychological mechanisms explaining how sleep problems can contribute to depression. Insomnia can affect depressive symptoms through maladaptive coping strategies [90], cognitive biases and repetitive negative thinking [91,92], rumination [93], increased emotional reactivity [94], pre-sleep arousal [95], and specific anxiety symptoms [96]. These factors can interact and increase vulnerability to both insomnia and depression. Addressing these factors, primarily through interventions targeting sleep problems and related psychological processes, may help reduce the risk and severity of both conditions, as well as prevent and treat them over academic time.

However, research indicated that different insomnia subtypes have distinct temporal effects on depression risk. Initial insomnia may take longer to manifest its impact, while nonrestorative sleep predicts depression across all follow-up periods. Middle and late insomnia both show weaker associations with depression onset [97]. Persistent and incident insomnia, as well as stable eveningness (a preference for later sleep times), are independently associated with increased depression risk in adolescents [98]. Relapse and persistent insomnia subgroups have the highest risk of developing depression, while remitted insomnia still carries an elevated risk compared to those without insomnia [99]. Therefore, future studies should control for the insomnia subtypes to fully explain the association between insomnia and depression in an academic population.

### 4.4. Mediating Role of Insomnia in the Relationship Between Problematic TikTok Use and Depression

The present study confirmed our hypothesis (H3) that insomnia mediates the relationship between PTTU and depression symptoms among university students. A similar mediation analysis was previously performed [77], but instead of TikTok, the general measurement of Internet use was examined in a sample of Chinese adolescents. Research consistently showed that students with higher smartphone or Internet addiction symptoms report more severe insomnia and depression symptoms [67,68,70,77,86]. Based on these studies, we assumed that problematic or excessive TikTok use is linked to increased rates of insomnia, which, in turn, increases depressive symptoms in university students. The assumption is confirmed in the study, explaining that insomnia plays a crucial role in the development of depression in those university students who excessively use TikTok.

University life, with its high academic pressures and social adjustments, can be a significant source of stress, which can contribute to both sleep disturbances and depression [6,21]. In particular, increased academic workload, added responsibilities, and the transition to a new environment can disrupt sleep patterns and exacerbate mental health challenges [75]. Also, adjusting to a new social environment, feeling isolated, or experiencing loneliness can negatively impact sleep and mental well-being [75]. Finally, among many factors contributing to worsened well-being is increased reliance on technology, especially late at night, which can disrupt sleep patterns and contribute to insomnia [5].

Insomnia and depression are common mental health challenges among university students. Insomnia is a strong predictor of future depression in university-aged populations. Students with persistent or relapsing insomnia are at much higher risk for developing depression over time compared to those without insomnia [21,22,23,93,95,99]. Multiple longitudinal studies demonstrate that insomnia is a significant predictor of future depression [95,97,100]. Frequent insomnia symptoms, including difficulties initiating or maintaining sleep and nonrestorative sleep, are associated with a moderately to substantially increased risk of developing depression over time. For example, individuals reporting insomnia symptoms five to seven nights per week had a 1.64-fold increased risk of incident depression treatment, and those with all four major insomnia symptoms had more than double the risk compared to those without insomnia [100]. Nonrestorative sleep, in particular, consistently predicted the onset of depression at 2-, 4-, and 6-year follow-ups [97]. Both new-onset and pre-existing insomnia symptoms predicted persistent depressive symptoms over 12 months, even after accounting for other risk factors [95]. Furthermore, the onset and maintenance of insomnia also partially mediate the relationship between anxiety and depressive symptoms over time, indicating that insomnia is both an outcome and a predictor within this network of symptoms [101].

It is also important to note that the relationship between insomnia and depression is often bidirectional: insomnia can predict depressive symptoms, and depression can also predict insomnia [93,95,96,101,102,103]. For instance, insomnia at baseline was related to new episodes of high depression at follow-up (odds ratio = 3.51), and depression at baseline predicted new cases of insomnia (odds ratio = 2.28) [103]. This reciprocal relationship suggests that insomnia and depression are intertwined over time, so both mental problems should be treated simultaneously.

### 4.5. Practical Implications

The study highlights the need for targeted interventions for specific user populations. The results of this study indicate that individuals at increased risk of PTTU may also have higher symptoms of insomnia and depression, so all three variables should be controlled for among university students. Targeting insomnia, particularly through interventions like cognitive behavioral therapy for insomnia, can significantly improve both sleep and depressive symptoms. Improvements in insomnia symptoms have been shown to mediate a large proportion (up to 87%) of the reduction in depressive symptoms following intervention [104]. Cognitive behavioral therapy for insomnia (CBT-I) is increasingly recognized as an effective treatment for individuals experiencing both insomnia and depression [105,106,107,108,109]. Research highlights its benefits not only for sleep but also for improving depressive symptoms, whether delivered alone, alongside antidepressants, or in digital formats. Improvements in insomnia symptoms during CBT-I are closely linked to subsequent remission of depression. Early reductions in insomnia severity predict later depression remission, suggesting that treating sleep problems can drive improvements in mood [110]. In particular, online CBT-I is effective for individuals with high depressive or anxiety symptoms, producing large and sustained reductions in both insomnia and comorbid symptoms over time [108]. Studies showed that even short, primary care-friendly versions of CBT-I can yield significant improvements in insomnia and moderate improvements in depression among patients with comorbid symptoms [109].

Sleep hygiene interventions are widely used to improve sleep quality among college students. Maintaining consistent sleep–wake schedules, avoiding arousing activities before bed, and creating a restful sleep environment is crucial. Improper sleep scheduling is a particularly strong predictor of insomnia severity in college students, highlighting the importance of regular sleep routines [111]. Effective interventions include brief, remotely delivered programs [112], behavior change techniques [113], comprehensive sleep management [114], and digital or text-based approaches [115,116]. These interventions not only enhance sleep hygiene and quality but can also reduce stress and improve overall well-being. In addition, consistent sleep routines and minimizing pre-bedtime arousal are especially important for lasting benefits. Removing electronic devices at bedtime and using sunrise alarm clocks can further improve sleep quality, reduce burnout, and lower perceived stress in students [117]. Also, brief online mindfulness and relaxation exercises supplemented with sleep strategies show promise for reducing stress and sleep difficulties [118].

Online interventions are increasingly used to address depression in university students. These interventions include online mindfulness-based interventions and cognitive behavioral therapy (CBT) programs [119,120,121,122,123], online guided self-help interventions, including those based on CBT and Acceptance and Commitment Therapy (ACT) [123,124], and self-guided mobile apps for depression, such as those incorporating CBT techniques [125]. These techniques are generally effective in reducing depressive symptoms, although the degree of benefit varies by intervention type and study quality. While the benefits are generally modest, these tools can reach students who might not otherwise seek help and can be integrated into broader mental health support strategies on campus.

For students experiencing problematic TikTok use (PTTU), participation in Internet and Technology Addicts Anonymous (ITAA) is advised [126]. ITAA employs a Twelve-Step fellowship based on the principles pioneered by Alcoholics Anonymous to assist individuals struggling with an addiction to social media that becomes compulsive and problematic. ITAA helps in achieving sustained liberation from self-destructive behaviors. During ITAA meetings, participants can share their experiences, strengths, and hopes through group and one-on-one interactions and engage in a recovery program grounded in the Twelve Steps of Alcoholics Anonymous. These meetings are free and confidential and include members of all ages, genders, and ethnicities from around the world in such languages as French, Spanish, Russian, German, Dutch, Hebrew, Arabic, and Polish. Digital literacy and responsible use of online platforms should also be promoted to prevent digital bullying and the spread of misinformation via TikTok or other social media [62].

### 4.6. Limitation of the Study

The study has several limitations that prevent the generalization of the results. First of all, the sample size is not large. Although the sample size is adequate for testing statistical hypotheses, it cannot be seen as representative of the general population of university students in Poland or other countries. Further studies should include more students recruited from all possible universities in the country. It is unclear whether the present results are specific to Polish culture or university students as a whole population independent of country and language. To answer this question, an intercultural study should be performed to compare the results between particular countries from various continents of the world. Also, in the present sample, women prevail over men. The study should be replicated in a more balanced gender sample in the future. Finally, the causal relationship between variables cannot be fully confirmed in the cross-sectional design of this study. Therefore, longitudinal research is required to verify the mediation model. In particular, a cross-lagged panel model (CLPM) would examine the variance of all variables at least three time points and could resolve the vicious circle problem that may exist in the bidirectional associations between PTTU, insomnia, and depression. Bias may also be a source of survey data that relies on self-reported data. Although these data seem crucial in psychological research, it would be interesting to conduct future studies using more objective measures of TikTok use (e.g., time used per day, per week, and per month) obtained directly from mobile phones, tablets, or other devices. Depression and insomnia were also not clinically diagnosed by a professional but were based on self-reported data, which may be biased by poor self-awareness, distorted self-image, cognitive biases, poor memory, stereotypes, low emotional intelligence, or a variety of other potential personality or contextual biases. The selection of subjects for the study is also subject to measurement error, as the participants were a conventional group of Internet users and primarily social media users, which may result in overestimated rates of all variables in the study. It is unknown how people who do not use social media would react. Therefore, paper-and-pencil studies should be conducted that could verify the current research results in a more heterogeneous sample. In this study, we did not control for the influence of other social media on participants. However, it is likely that students use several social media simultaneously (e.g., Facebook, Instagram, YouTube, TikTok, etc.). Future studies should be conducted that can separate the influence of individual social media to clearly answer the question of how much TikTok explains the variance in mental health disorders. Finally, numerous factors, including age, gender, life stress, substance use (encompassing alcohol, smoking, and illicit drugs), and comorbid psychiatric or health-related conditions, may confound the relationships between study variables. Consequently, it is imperative to control for a greater number of variables in replication studies.

## 5. Conclusions

The study revealed no significant differences in problematic TikTok use (PTTU), insomnia, and depression based on gender or academic faculty. These findings suggest that university students constitute a homogeneous group, with these three variables influencing their mental health independently of gender and field of study. All variables are interrelated, with insomnia playing a mediating role in the relationships between PTTU and depression. The study demonstrated a mechanism by which PTTU contributes to the exacerbation of depression symptoms through sleep disturbances. Therefore, prevention and intervention efforts should primarily target university students exhibiting symptoms of insomnia. Given that excessive TikTok use can exacerbate sleep disturbances, it should also be considered in campus-based prevention and intervention strategies. Due to the strong correlation between insomnia and depression, both conditions should be monitored and addressed within the academic population, independent of gender and faculty of university students.

## Figures and Tables

**Table 1:** Gender differences in depression, insomnia, and problematic TikTok use among university students (*N* = 173).

Variable	Men (*n* = 45)		Women (*n* = 128)		*t*(171)	*p*	*d*
	*M*	*SD*	*M*	*SD*			
Depression	8.71	5.67	9.61	5.51	−0.93	0.352	−0.16
Insomnia	8.31	4.54	8.16	4.38	0.19	0.848	0.03
PTTU	12.18	6.60	13.20	6.08	−0.95	0.342	−0.17

**Table 2:** Faculty differences in depression, insomnia, and problematic TikTok use among university students (*N* = 173).

Variable	Non-Medical (*n* = 89)		Medical (*n* = 84)		*t*(171)	*p*	*d*
	*M*	*SD*	*M*	*SD*			
Depression	9.58	5.87	9.16	5.21	0.51	0.612	0.08
Insomnia	8.29	4.89	8.11	3.86	0.28	0.783	0.04
PTTU	13.08	6.44	12.79	6.00	0.31	0.758	0.05

**Table 3:** Differences in insomnia and depression symptoms between university students using TikTok excessively and non-problematically (*N* = 173).

Variable	Non-PTTU (*n* = 134)		PTTU (*n* = 39)		*t*(171)	*p*	*d*
	*M*	*SD*	*M*	*SD*			
Insomnia	7.70	4.33	9.92	4.29	−2.83	0.005	−0.51
Depression	8.86	5.56	11.15	5.19	−2.30	0.022	−0.42

Note. PTTU = problematic TikTok use.

**Table 4:** Mediating effects of insomnia on the relationships between problematic TikTok use and depression (*N* = 173).

				95% CI				
Type	Effect	*b*	*SEb*	LL	UL	β	*z*	*p*
Indirect	PTTU ⇒ AIS ⇒ PHQ	0.16	0.05	0.08	0.25	0.18	3.59	<0.001
Component	PTTU ⇒ AIS	0.20	0.05	0.09	0.30	0.28	3.78	<0.001
	AIS ⇒ PHQ	0.82	0.07	0.67	0.97	0.66	11.49	<0.001
Direct	PTTU ⇒ PHQ	0.10	0.05	−0.03	0.20	0.11	1.95	0.052
Total	PTTU ⇒ PHQ	0.26	0.07	0.13	0.38	0.29	4.00	<0.001

Note. PTTU = problematic TikTok use, AIS = Athens Insomnia Scale for measuring insomnia symptoms, PHQ = Patient Health Questionnaire for assessing depression symptoms, LL = lower level, and UL = upper level. Confidence intervals (CI) computed with bias-corrected bootstrap method.

## Data Availability

Raw data supporting reported results can be found at Mendeley Data [127].

## Abbreviations

The following abbreviations are used in this manuscript:

AIS	Athens Insomnia Scale
BFAS	Bergen Facebook Addiction Scale
CI	Confidence interval
ICD	International Classification of Diseases
LL	Lower level
PHQ	Patient Health Questionnaire
PTTU	Problematic TikTok use
UL	Upper level
